# A meta‐analysis and systematic review of interventions to prevent or treat cognitive decline related to Alzheimer's disease in adults with Down syndrome

**DOI:** 10.1002/alz.70471

**Published:** 2025-07-25

**Authors:** Emily E. Munn, Anna Montelongo, Viraj K. Patel, Jill C. Fodstad, Mary R. Ciccarelli, Lauren T. Ptomey, Melissa M. Pangelinan

**Affiliations:** ^1^ Kinesiology Department School of Public Health Indiana University Bloomington Bloomington Indiana USA; ^2^ Indiana University School of Medicine Indianapolis Indiana USA; ^3^ Department of Psychiatry Indiana University School of Medicine Indianapolis Indiana USA; ^4^ Department of Pediatrics Indiana University School of Medicine Indianapolis Indiana USA; ^5^ Department of Internal Medicine University of Kansas Medical Center Kansas City Kansas USA; ^6^ Indiana School of Medicine Stark Neuroscience Research Institute Indianapolis Indiana USA

**Keywords:** aging, dementia, prevention, treatment, trisomy 21

## Abstract

Down syndrome (DS) is a leading genetic risk factor for Alzheimer's disease (AD). Although prevention and treatment of AD are well documented for those without DS, less is known about individuals with DS. This review evaluates the efficacy of AD and dementia interventions in people with DS. It includes 26 articles with 1417 participants ages 18–69 in the qualitative synthesis and 17 articles with 1118 participants in the meta‐analysis. Four types of interventions demonstrated small‐to‐moderate effects: pharmacological (*n* = 19), exercise (*n* = 5), environmental (*n *= 1), and cognitive training (*n *= 1). Significant treatment effects were observed across interventions (*t*(66) = 4.67, *p* < 0.0001, *d* = 0.29, 95% confidence interval [CI]: 0.16–0.40); however, high heterogeneity (*I^2^* = 93.8% CI = 92.8–94.7) was noted, which was not explained by intervention type or outcome. The meta‐analysis indicates that interventions enhance AD‐related cognitive and behavioral outcomes, emphasizing the need for further research to address heterogeneity. This review was registered with PROSPERO: CRD42024567026.

**Highlights:**

- This study is the first to comprehensively review both pharmacological and non‐pharmacological interventions for Alzheimer's disease (AD) in individuals with Down syndrome (DS), extending previous reviews by including a meta‐analysis and examining key mediating variables.
- Donepezil, the most studied pharmacological treatment, showed significant cognitive and behavioral improvements in individuals with DS, especially with longer treatment periods. However, further trials are needed to explore its efficacy in combination with non‐pharmacological interventions.
- All seven non‐pharmacological studies reported significant improvement, suggesting that even small doses of exercise and cognitive training can be effective and feasible for individuals with DS. Online formats may enhance scalability and reduce barriers to participation.
- Significant heterogeneity in cognitive assessments across studies highlights the need for standardized, sensitive assessments to enable meaningful comparisons of intervention effects.
- Additional studies are required to determine the long‐term efficacy of pharmacological interventions like Donepezil and to assess the sustained impact of non‐pharmacological interventions on key AD‐related cognitive domains such as memory, language, and executive function.

## 1 INTRODUCTION

The population of individuals with Down syndrome (DS) living in the United States has increased substantially over the last 60 years, from ≈50,000 to ≈200,000 people, due to medical advancements, social changes, and transition to community living.^1^ Life expectancy has been estimated to be ≈60 years.^2^, ^3^, ^4^ As such, a growing number of adults with DS will experience aging‐related diseases, including Alzheimer's disease (AD). Indeed, the incidence of clinical symptoms of AD increases dramatically from 8% to 80% between the ages of 40 and 54 years in persons with DS.^4^, ^5^, ^6^, ^7^ AD is associated with an estimated 70% of deaths in persons with DS,^8^, ^9^ due in part to the triplication of the amyloid precursor protein (*APP*) gene on chromosome 21.^8^ Thus, identifying evidence‐based prevention and treatment for persons with DS is critical to attenuate the significant burden of AD.

Few clinical trials of AD treatment have included individuals with DS, so the efficacy of these treatments for this population is not well known.^10^ In contrast, in people without DS, both pharmacological and non‐pharmacological prevention and treatment strategies can slow the progression of AD.^11^, ^12^, ^13^, ^14^, ^15^, ^16^, ^17^ Published reviews of interventions and treatments for AD in individuals without DS include pharmacological treatments,^11^, ^15^ cognitive interventions,^14^, ^16^ physical activity interventions,^13^, ^16^ and other therapeutic interventions.^12^, ^17^ Proposed pharmacological treatments include anti‐amyloid therapy,^16^ anti‐tau therapy,^16^ and anti‐neuroinflammatory^16^ therapy. Common cognitive interventions include completing prescribed tasks or games aimed at improving AD‐related cognitive functions (including memory and executive function). Common physical activity interventions involved walking, dancing, strength training, and functional fitness. Taken together, pharmacological, cognitive, and exercise treatments are effective in delaying the onset of AD, reducing symptoms of AD, or attenuating AD‐related cognitive decline in persons without DS. Although there are no treatments for the prevention of AD in the general population, there are currently two U.S. Food and Drug Administration (FDA)–approved treatments for early AD: donanemab and lecanemab. Individuals with DS are not precluded from using these treatments. However, safety studies are needed to document their efficacy and safety for persons with DS.^18^

Previous systematic reviews assessing AD treatment in DS focused primarily on pharmacological interventions, including donepezil, simvastatin, rivastigmine, and memantine.^19^, ^20^ Of these pharmacological interventions, there is preliminary evidence that simvastatin is associated with improved cognitive outcomes.^20^ However, challenges in assessing changes in cognitive function in individuals with DS have been noted.^21^, ^22^ For example, depending on the level of intellectual disability, assessment floor effects may preclude precise measurement of cognitive changes.^22^ Patients and caregivers may not be aware of the earlier age of symptom manifestation (compared to non‐DS populations), which may lead to delayed symptom identification or diagnosis until later in the disease progression. Heterogeneity in the cognitive assessment used as a primary outcome in research also makes direct comparisons across studies difficult. Given these challenges, Rafii^21^ called for greater consistency in study design, inclusion criteria (including standardized AD diagnostic assessments), harmonized outcomes assessments, and multi‐site studies to increase reach and generalizability. In addition, the onset of symptoms occurs much earlier than in the general population; therefore, there is a need for even earlier intervention. Finally, there is a need to use common cognitive assessments to enable direct comparison across studies.^19^, ^21^, ^22^

To our knowledge, no systematic reviews have comprehensively assessed pharmacological and non‐pharmacological interventions for preventing and treating AD in individuals with DS. Thus, the purpose of this systematic review and meta‐analysis is to address this knowledge gap. The first aim was to summarize the efficacy of different types of interventions. The second aim was to conduct a meta‐regression to quantify the effects of prevention and treatment that differ by intervention type (pharmacological vs non‐pharmacological) and cognition‐based outcome assessment.

## 2 METHODS

This study followed the 2020 Preffered Reporting Items for Systematic Reviews and Meta‐Analyses (PRISMA) review guidelines and was registered with PROSPERO (International prospective for register od systematic reviews; # CRD42024567026) before data extraction.^23^ Three authors (E.M., A.C., and V.K.) conducted the abstract review, full‐text review, and final article selection for the qualitative and quantitative synthesis.

### 2.1 Data sources

The following databases were queried: Medline, Academic Search Premier, CINAHL, Health Source: Nursing/Academic Edition, Eric, and SPORTDiscus. The following search terms were used: (Down syndrome OR Downs) AND (Alzheimer's OR dementia OR cognitive impairment) AND (intervention OR treatment OR program) AND (adult). Two systematic reviews were also examined^19^, ^20^ for additional articles.

### 2.2 Inclusion and exclusion criteria

This review aimed to identify interventions targeting AD, dementia, or related cognitive outcomes in individuals with DS. It was limited to articles with a program, intervention, or treatment. Studies were included if they were in English, were peer‐reviewed, included adults 18 years or older only diagnosed with Down syndrome, and were published by December 1, 2023. In addition, as this project focused on AD, only articles with dependent variables related to AD, dementia, or related cognitive and behavioral outcomes were included. Studies were excluded if they did not meet the inclusion criteria or were mouse models.

### 2.3 Data extraction

Three authors (E.M., A.M., and V.P.) independently reviewed the titles and abstracts. Then three reviewing authors met, reread, and discussed the articles where they disagreed. Following abstract review and discussiong, the three authors (E.M., A.M., and V.P.) indepentantly reviewed the full text independently. After indepentant review, the three reviewing authors met again to reread, and discussed the articles where they disagreed for full inclusion. All reviewing authors then agreed on the final articles. One additional article was identified during the review process and added.

Following final review, the three reviewing authors pulled primary diagnosis, secondary diagnosis, participant number, number/type of groups, ages, type of intervention, drug category (if applicable), research design, dose (times/minutes a day, etc.), medication dose (if applicable), length of study, primary cognition‐based dependent variable (all variables if not noted), summary of the key findings, means, standard deviations (SDs; requested if not reported), effect size (if report), and any other reported statistics. The first author merged the three reports and confirmed that everyone's matched.

Additional screening was conducted for inclusion in the meta‐analysis: (1) the study reported the means and SDs to calculate between‐group effect sizes, and (2) the study included two or more groups (case studies were excluded). The corresponding author was contacted if these data were not included in the published article.

Data for the primary cognitive outcomes were extracted for each article. When multiple primary outcomes were identified, all were included. When no primary outcome was listed, all cognitive outcomes were included. Studies were categorized as pharmacological or non‐pharmacological (exercise, environmental, or cognitive training). Outcomes were categorized as adaptive behavior, dementia or AD symptoms, executive functioning and cognitive flexibility, language, memory, sensorimotor, processing speed, or visuospatial.

### 2.4 Data analysis and synthesis

Baseline and post‐intervention means and SDs, or reported effect sizes (Cohen's *d*), were used to compute a standard mean difference (Cohen's *d*) between the intervention and control group using the Metaphor and dmeter packages in *R* (R2024, version 2024.09.0+375). Heterogeneity was assessed with the *I* ^2^ statistic. Meta regressions were used to assess the effects of intervention type (pharmacological or non‐pharmacological), variable type (adaptive behavior, dementia/AD scale, executive functioning and cognitive flexibility, language, memory, processing speed, sensorimotor, and visuospatial processing), dose, risk of bias, and drug category (for the pharmacological interventions only). Effect size was assessed through given mean differences or calculated mean differences from the means and SDs in the form of Cohen's *d*. The level of significance was set at *p *< 0.05 for all analyses.

### 2.5 Assessment of bias

Three researchers (E.M., V.P., A.M.) independently assessed the risk of bias using two tools: Cochrane Risk of Bias 2 (RoB 2) for randomized control trials^24^ and Risk of Bias In Non‐Randomized Studios of Interventions (ROBINS‐I) for non‐randomized control trials.^25^ All disagreements were settled through group discussion with the three senior authors. Interrater reliability was 80%. The risk of bias was included as a variable in the meta‐regression.

## 3 RESULTS

**FIGURE 1:**
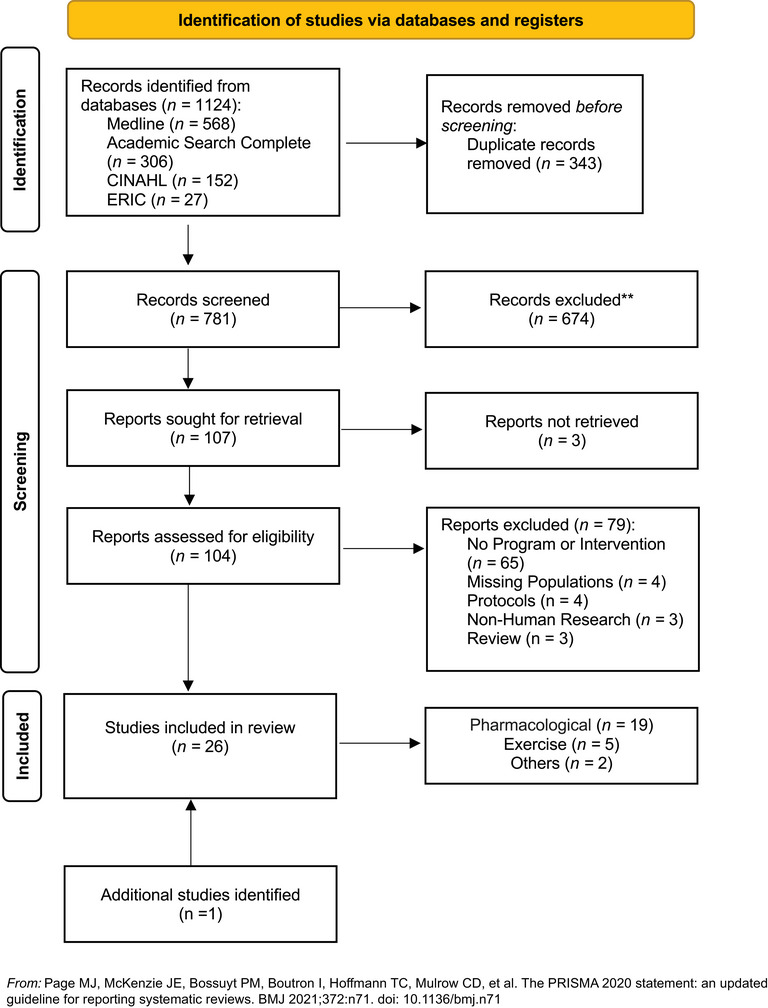
PRISMA flowchart of the article selection process.

Figure 1 depicts the PRISMA flow diagram to illustrate the stages of identification and review. The initial search of articles was conducted on December 1, 2023—returning 1124 articles. After duplicates were removed (*n* = 343 articles), 781 records were screened for inclusion by three authors (E.M., A.M., and V.P.) independently. A total of 107 abstracts were then included in a full‐text review. However, three of these articles had only the abstract in English, leaving 104 for full‐text review. Eighty percent agreement occurred at the abstract phase. The three reviewing authors met, reread, and discussed the articles to come to an agreement. After discussion, all authors agreed on the 104 articles to move to full text review. After full‐text review, a total of 79 of these articles were excluded for the following reasons: no program or intervention (*n* = 65), missing DS or AD dementia information (*n* = 4), description of a protocol (*n* = 4), non‐human research (*n* = 3), and review article (*n* = 3). Ninety percent of agreement occurred at the full‐text phase. The three reviewing authors met, reread, and discussed the articles about which they disagreed. All reviewing authors then agreed on the final 25 articles. An additional article was identified, reviewed, and added during the review process. A total of 26 articles are included and summarized in the qualitative synthesis.

For the quantitative synthesis, 17 articles included the necessary information. The corresponding author was contacted if the necessary data were not included in the published manuscript (*n* = 5). Eight articles were excluded from the meta‐analysis for missing data (*n *= 5) and case studies (*n *= 3). Seventeen articles were included in the meta‐analysis (quantitative synthesis).

The 26 articles included in the qualitative synthesis were published between 1991 and 2023.^26^, ^27^, ^28^, ^29^, ^30^, ^31^, ^32^, ^33^, ^34^, ^35^, ^36^, ^37^, ^38^, ^39^, ^40^, ^41^, ^42^, ^43^, ^44^, ^45^, ^46^, ^47^, ^48^, ^49^, ^50^, ^51^ Four types of interventions were identified: pharmacological (*n = *19), exercise (*n = *5), environmental (*n = *1), and cognitive training (*n = *1). A total of 1436 participants were included in the studies: pharmacological (*n = *1221), exercise (*n = *115), environmental (*n = *60), and cognitive training (*n = *40). Study design included randomized control trials (RCT; *n = *14), non‐randomized control trials (NRCT; *n = *7), and case studies (*n = *3), two‐group pre‐post (2G; *n = *1), quasi‐experimental pre‐post (QE; *n = *1). Ten studies included multiple primary outcomes. In addition, three studies included multiple intervention groups; all intervention groups were included in the quantitative analysis, with the different groups nested within the study. As such, 58 variables of cognitive outcomes related to AD or dementia were assessed.

### 3.1 Risk of bias

**FIGURE 2:**
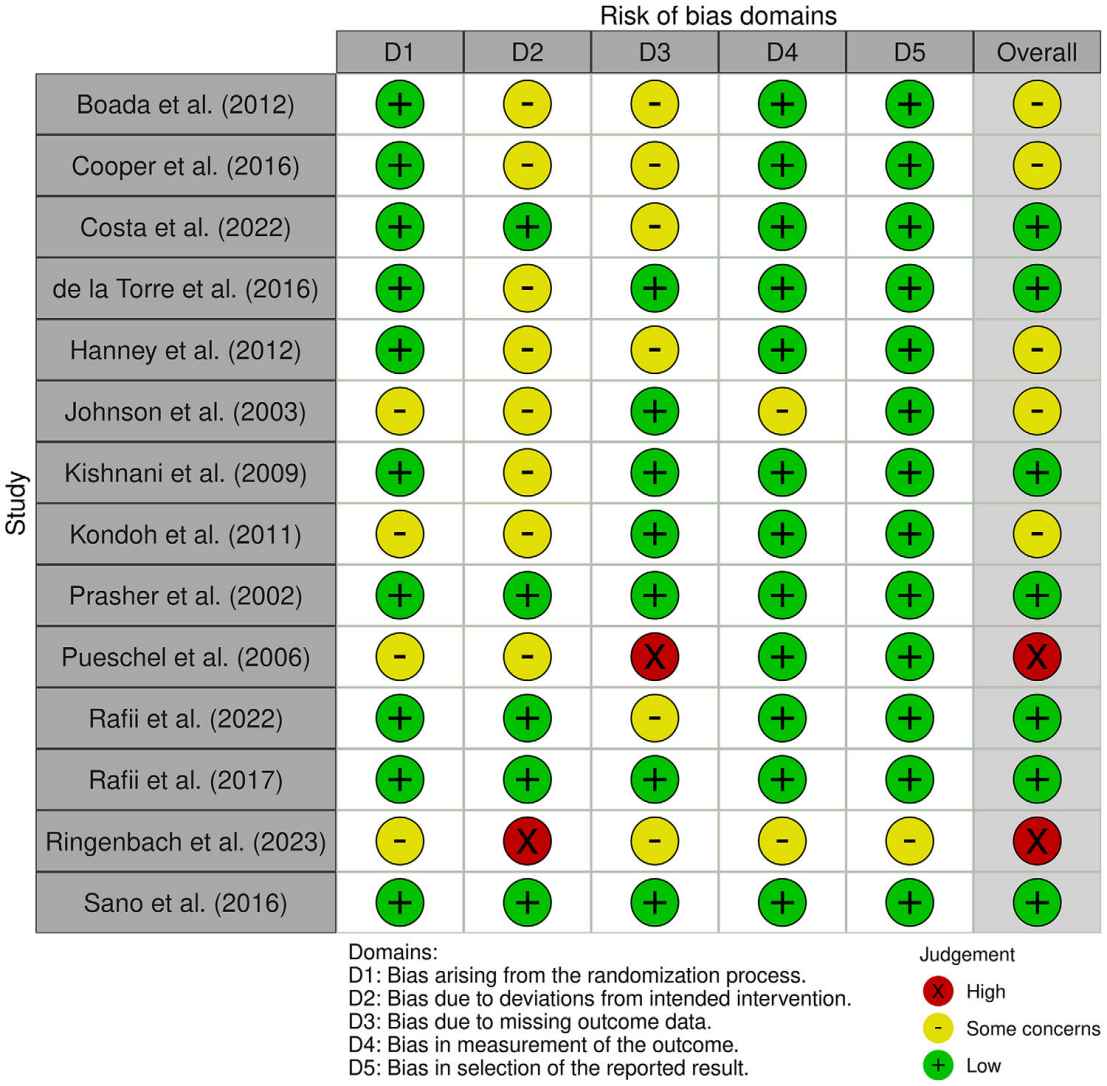
Risk of bias for randomized control trials—RoB‐2.

Figure 2 presents the risk of bias assessment for the 14 randomized control trials using the RoB2.^24^ Two studies were rated at a high risk of bias.^46^, ^48^ Five studies were rated as having a moderate risk of bias.^26^, ^30^, ^35^, ^36^, ^39^ Six studies were rated a low risk of bias.^31^, ^44^, ^47^, ^49^, ^51^, ^52^, ^53^ Thirty‐one percent of the studies had a bias that occurred during the randomization process. Bias was due to: 31% randomization process (Domain 1), 69% deviations from the intended intervention (Domain 2), 46% missing data (Domain 3), 15% measurement of the outcome (Domain 4), and 8% reporting results (Domain 5).

**FIGURE 3:**
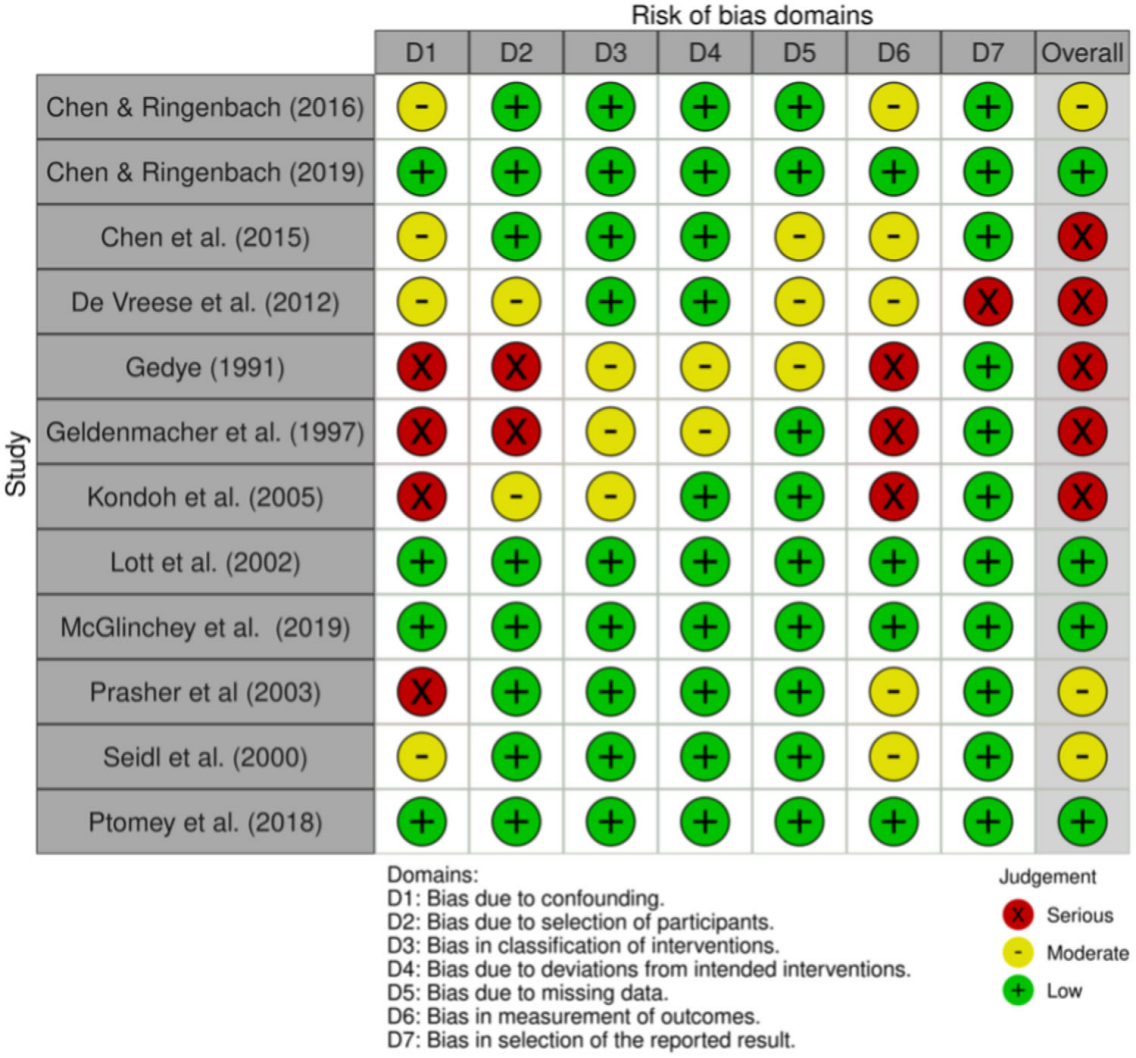
Risk of bias for non‐randomized control trials—ROBINS‐I.

Figure 3 presents the risk of bias for 12 non‐randomized control trials, which were assessed using the ROBINS‐I (Higgins et al., 2024). Five studies had a serious risk of bias.^28^, ^32^, ^33^, ^34^, ^38^ Three studies had a moderate risk of bias.^27^, ^42^, ^50^ Four studies had a low risk of bias.^29^, ^40^, ^41^, ^45^ Risk of bias was due to 67% confounding factors (D1), 33% participant selection (D2), 25% classification of interventions (D3), 17% deviations from the intended intervention (D4), 25% missing data (D5), 67% measurement outcomes (D6), and 8% reporting result (D7).

### 3.2 Pharmacological

The 18 pharmacological studies included a total of 1205 individuals and evaluated 9 different drugs. The number of participants ranged from 1^33^ to 337.^54^ This group of studies consisted of RCT (*n = *13), NRCT (*n = *3), and Case Study (*n = *3). The drugs/supplements used were donepezil (*n *= 7), memantine (*n =* 3), epigallocatechin gallate (EGCG; *n = *1), scylla‐inositol (ELND005; *n = *1), selective serotonin reuptake inhibitors (SSRIs; *n = *1), nicotine (*n = *1), acetylcarnitine (*n = *1), simvastatin (*n = *1), trazodone (*n = *1), vitamin E (*n = *1), and ACI‐24 (*n = *1). Eleven articles focused on prevention through improving or protecting from loss of cognitive functioning. Eight articles including treatments of diagnosed AD/dementia. The study length ranged from a one‐time dose on a single day to 2190 doses across 3 years. Table 1 provides details for the pharmacological studies.

**TABLE 1:** Pharmacological intervention.

Citations (APA)	Risk of bias	Treatment or prevention	Participants	Mean ages (SD)	Medication dose	Research design	Study duration	Dependent variables	Results
**Acetylcarnitine (symptom modifying)**									
Pueschel (2006)^46^	High	Prevention	Exp: *n* = 20 Placebo: *n *= 20	Exp: *M = *20.2 (19.3–22.8) Placebo: *M* = 21.5 (19.9–23.2)	10 mg/kg, 3x/day for month 1 20 mg/kg, 3x/day for month 2 30 mg/kg, 3x/day for months 3–6	RCT	9 months: 6 months and a “wash out” period of 3 months	Stanford‐Binet Intelligence Scale Hiskey–Nebraska visual attention span and matching familiar figure test Mazes and Coding Subjects Riddles Subtest Daily Living Skills subtest Child Behavioral Checklist	No difference between groups for intelligence, attention, visuomotor performance, verbal comprehension, daily living skills, or social/behavioral functions
**DONEPEZIL (disease modifying)**									
Johnson et al. (2003)^36^	Medium	Prevention	Exp: *n *= 9 Placebo: *n* = 9	Exp: *M* = 29.5 (3.1) Placebo: *M* = 24.7 (2.6)	5 mg/day for first 6 weeks 10 mg/day for next 6 weeks	RCT	12 weeks	Severe cognitive impairment profile (SCIP) Scales of Independent Behavior‐Revised	Exp: improvement in the language subtest of the SCIP. No difference in memory, attention, or total score of the SCIP or caregiver ratings on Scales of Independent Behavior‐Revised Placebo: No difference from baseline to 3 months for any measure
Kondoh et al. (2005)^38^	High	Treatment	Exp: *n *= 2	Case 1: 38 years old Case 2: 22 years old	Case 1: 5 mg/day for 1 month, 3 mg/day for next 3 years Case 2: 3 mg/day for 1.75 years	Case	Case 1: 3 years Case 2: 21 months	Adaptive Behavior Scale	Case 1: Increase from baseline to 3 years for the adaptive behavioral scale (independent function, domestic ability, vocational ability, responsibility) Case 2: Increase from baseline to 21 months for the adaptive behavioral scale (spontaneity, vocabulary, speech, communication skills, independent function, physical development, language development, self‐direction, responsibility, socialization)
Kondoh et al. (2011)^39^	Medium	Prevention	Exp: *n* = 11 Placebo: *n *= 10	M *=* 45.6 (32‐58)	3 mg/day for 24 weeks	RCT	24 weeks	International Classification of Functioning, Disability and Health (ICF) scales	Exp: Increase from baseline to 24 weeks for ICF scales (global mental health, specific mental functions, voice, and speech functions) Placebo: No difference from baseline to 24 weeks for ICF scales
Lott et al. (2002)^40^	Low	Treatment	Exp: *n *= 9 Placebo: *n *= 6	Exp: *M *= 52.3 Placebo: *M* = 52.5	5 mg for a mean of 50.9 days (± 46.6) 10 mg for a mean of 78.2 days (± 59.5)	NRCT, 2 group Pre‐Post	5 months	Down syndrome dementia scale (DSDS)	Exp: Significant improvement from baseline to 5 months for the DSDS compared to the control group Placebo: No difference from baseline to 5 months
Prasher et al. (2003)^42^	Medium	Treatment	Exp: *n *= 6 Placebo: *n* = 7	Exp: *M* = 51.0 (8.9) Placebo: *M* = 56.0 (4.8)	2.5 mg‐10 mg/day	NRCT, 2 group Pre‐Post	104 weeks	Dementia scale for Mentally Retarded Person (DMR) Adaptive behavior scale (ABS) Neuropsychiatric inventory (NPI) Severe impairment battery (SIB)	Exp: Decrease from baseline to 24 weeks in global functioning on the DMR, 3.8% decrease in ABS. No difference in NPI or SIB. Placebo: Decrease from baseline to 24 weeks in global functioning on the DMR and ABS. No difference in NPI or SIB
Prasher et al. (2002).^44^	Low	Treatment	Exp: *n* = 16 Placebo: *n* = 15	Exp: *M* = 53.06 (8.03) Placebo: *M *= 55.07 (4.60)	5 mg/day for 4 weeks 10 mg/day for 20 weeks	RCT	24 weeks	Dementia Scale for Mentally Retarded Persons (DMR) Severe Impairment Battery	Exp: Slight improvements from baseline to 24 weeks on the DMR Placebo: Slight improvements from baseline to 24 weeks on the DMR. No group difference in the change from baseline to 24 weeks. No improvements in SIB
Kishnani et al. (2009)^53^	Medium	Prevention	Exp: *n *= 62 Placebo: *n *= 61	Exp: *M* = 24.2 (5.1) Placebo: *M* = 26.0 (5.5)	5 mg/day for 6 weeks 10 mg/day for 6 weeks	RCT	12 weeks	Severe Impairment Battery (SIB)	Double‐blind phase: Exp: No difference from baseline to 12 weeks for cognitive function on the SIB Placebo: No difference from baseline to 12 weeks for cognitive function on the SIB
**EPIGALLOCATECHIN GALLATE, ANTIOXIDANT (disease modifying)**									
de la Torre et al. (2016)^31^	Low	Prevention	Exp: *n *= 43 Placebo: *n* = 44	Exp: *M* = 23.1 (3.6) Placebo: *M* = 23.4 (4.9)	600 mg/day EGCG (for participants 50‐75 kg) 800 mg/day ECGC (for participants 75–100 kg)	RCT	12 months	Pattern Recognition Memory Test Cats & Dogs Stroop Task Adaptive Behavior Assessment System 2	Exp: preservation of memory (pattern recognition memory test), improvement in executive functioning (switch condition of Cat and Dog Stroop), improvement in the ABAS‐II Placebo: more deterioration of memory, no changes to executive functioning and ABAS‐II
**SCYLLO‐INOSITOL (disease modifying)**									
Rafii et al. (2017)^47^	Low	Prevention	2x/day: *n* = 12 1x/day: *n* = 4 Placebo: *n* = 6	2x/day: *M* = 26.3 (4.43) 1x/day: *M* = 27.8 (4.03) Placebo: *M* = 30.0 (5.93)	BID: ELND005 250 mg, 2x/day QD: ELND005 250 mg, 2x/day	RCT	4 Weeks	Neuropsychiatric inventory Rapid assessment for DD (RADD) Block Design Subtest of Wechsler Adult Intellect Scale Vineland Adaptive Behavior Scale IV	No difference from baseline to 4 weeks any measure
**FLUOXETINE, SERTRALINE, PAROXETINE (symptom modifying)**									
Geldmacher et al. (1997)^34^	High	Treatment	*n* = 6	*M* = 44.3 (23‐63)	Varied based on SSRI and other medications use	Case	Unclear	Behavior Test	Case 1: Increased social interaction and independent ADL Case 2: Increased social interaction, decreased outbursts Case 3: Increased toileting, decreased disruptive behavior, increased forgetfulness Case 4: Increased social interaction, decreased hallucinations, increased compulsive behaviors Case 5: Decreased compulsive behavior, decreased agitation Case 6: Increased social interaction, decreased negative behaviors
**MEMANTINE (disease modifying)**									
Boada et al. (2012)^26^	Medium	Prevention	Exp: *n *= 19 Placebo: *n *= 19	Exp: *M* = 23.27 (3.52) Placebo: *M* = 22.60 (4.01)	5 mg, 1x/day for week 1 5 mg, 2x/day for week 2 5 mg and 10 mg for week 3 10 mg, 2x/day for weeks 4–16	RCT	16 Weeks	CANTAB Paired Associated Learning Stages	Exp: No difference in CANTAB paired associates learning stages.
Costa et al. (2022)^52^	Low	Prevention	Exp: *n* = 81 Placebo: *n *= 79	Exp: *M* = 20.4 (4.7) Placebo: *M* = 20.3 (4.2)	5 mg, 1x/day for week 1 5 mg, 2x/day for week 2 7.5 mg, 2x/day for week 3 10 mg, 2x/day for weeks 4–16	RCT	16 weeks	California Verbal Learning Test—Second Edition Go‐No‐Go Scales of Independent Behavior (SIB) Paired Associates Learning (PAL Peabody Picture Vocabulary Test (PPVT)	Exp: No difference in CVLT‐II, Go‐No Go task, PAL, PPVT or SIB
Hanney et al. (2012)^35^	Medium	Treatment	Exp: *n *= 88 Placebo: *n* = 85	Exp: *M* = 51.7 (7.3) Placebo: M = 51.0 (6.9)	5 mg/day for 8 weeks 10 mg/day for 44 weeks	RCT	52 Weeks	Down Syndrome Attention, Memory, and Executive Function Scales (DAMES) Adaptive Behavioral Scale	Exp: No significant difference in cognitive functioning, adaptive behavior, memory, and attention.
**NICOTINE (Symptom modifying)**									
Seidl et al. (2000)^50^	Medium	Prevention	DS: *n *= 5 non‐DS: *n* = 5	DS: 18‐34 non‐DS: 20‐34	5 mg nicotine patch single application for 2 hours	NRCT, 2 group Pre‐Post	2 Hours	Digit‐Symbol Subtest of Neuropsychological Test	Exp: Significant improvements in Digit‐symbol subtest of Neuropsychological Test
**SIMVASTATIN (symptom modifying)**									
Cooper et al. (2016)^30^	Medium	Prevention	Exp: *n* = 10 Placebo: *n* = 11	Exp: *M* = 54.68 (3.10) Placebo: *M* = 53.67 (3.16)	40 mg/day for 12 months	RCT	12 Months	Selective Attention Cancellation Test Memory Object Test Tower of London (Revised for Learning Disabilities) Cat & Dogs Switching Condition Category Fluency Test Pattern Recognition Memory % Correct Cued Recall Test Story Recall (adapted from Riverhead Behavior Memory for Children)	Exp: Significant improvements in memory in the Neuropsychological Assessment of Dementia in Individuals with Disabilities test. No other significant changes. Placebo: no significant changes.
**TRAZODONE (symptom modifying)**									
Gedye (1991)^33^	High	Treatment	*n *= 1	58 years old	100 mg/day for 15 days tapered for 5 days until no treatment phase (15 days) 100 mg/day for 5 days 200 mg/day for 45 days	Case	30 Days	Aggression (number of minutes of aggression)	Case: The number of minutes of aggression significantly decreased from the baseline phase to the treatment phase. Increase during the washout phase and decrease during the second treatmen*t* phase.
**VITAMIN E (symptom modifying)**									
Sano et al. (2016)^54^	Low	Treatment	Exp: *n* = 168 Placebo: *n* = 169	Exp: *M* = 54.82 (4.75) Placebo: *M* = 54.09 (3.74)	670 mg, 2x/day for 3 years	RCT	3 Years	Fluid Recall	Exp: No significant differences Placebo: No significant changes
**ACI‐24 Vaccine (Disease modifying)**									
Rafii er al., 2022^51^	Low	Prevention	Exp 1: *n* = 6 Exp 2: *n* = 6 Placebo: *n* = 4	Exp 1: *M* = 33.5 (4.6) Exp 2: *M* = 31.5 (4.9) Placebo: *n* = 33.0(4.2)	Exp 1: 7 doses of 300 µg across 48 weeks Exp 2: 7 doses or 1000 µg across 48 weeks	RCT	96 Weeks	Reaction Time Paired Associated Learning Scale Adaptive Behavior	Exp: No significant differences in reaction time, memory, or adaptive behavior

Abbreviations: ABS, Adaptive Behavior Scale; ABAS‐II, Adaptive Behavior Assessment System 2; Case, case studies; CVLT‐II, California Verbal Learning Test Second Edition; DAMES, Down Syndrome Attention, Memory, & Executive Function Scales; DMR, Dementia Scale for Mentally Retarded Person; DSDS, Down Syndrome Dementia Scale; Exp, experimental group; ICF, International Classification of Functioning, Disability and Health; kg, kilograms; mg, milligrams; NPI, Neuopsychiatric Inventory; NRCT, non‐randomized controlled trial; PAL, Paired Associates Learning; RCT, randomized controlled trial; SCIP, Severe Cognitive Impairment Profile; SD, standard deviation; SIB, Severe Impairment Battery.

### 3.3 Acetylcarnitine

One study examined the effects of acetylcarnitine on preventing decline in intelligence, attention, visuomotor performance, verbal comprehension, daily living skills, or social/behavioral functions.^46^ Acetylcarnitine is a symptom‐modifying drug. Participants, ages 19.3–22.8 in the experimental group, received 10 mg/kg/day of acetylcarnitine during the first month, 20 mg/kg/day during Month 2, and 30 mg/kg/day for Months 3–6. In the end, there were no significant changes in intelligence, attention, visuomotor performance, verbal comprehension, daily living skills, or social/behavioral functions for the experimental groups.

### 3.4 Donepezil

Donepezil is a disease‐modifying treatment^55^ that works as a cholinesterase inhibitor.^53^ It has been approved to treat AD.^39^ Donepezil inhibits acetylcholinesterase, which is responsible for degrading acetylcholine.^53^ Seven studies examined the effects of donepezil on cognitive function,^36^, ^38^, ^39^, ^40^, ^42^, ^44^, ^53^ with three studies examining more than one primary outcome (dementia questionnaire, adaptive behavior scale, global health functioning). Three studies^36^, ^39^, ^53^ examined the prevention of symptoms in participants ages 18–58. Four studies^38^, ^40^, ^42^, ^44^ examined the treatment of AD in participants ages 22–60+. These studies ranged in length from 3 months^36^ to 3 years,^38^ with an average duration of about 51 weeks. The dose ranges from 2.5^44^ to 10 mg/day.^36^, ^40^, ^42^, ^44^

Four studies^38^, ^39^, ^40^, ^42^ reported significant improvements in cognition or adaptive skills outcomes. Three studies^36^, ^44^, ^53^ reported mixed results. Two reported either improvements or less decline on the Adaptive Behavior Scale.^38^, ^42^ Two studies reported less decline on the Dementia Questionaire for Mental Retarded Persons (DMR) for the experimental over the placebo control group.^42^, ^44^ Two studies reported no changes on the Severe Impairment Battery for both the experimental or placebo control groups.^44^, ^53^ Lott et al.^40^ reported significant improvements on the DSDS for the experimental groups, while the placebo control group saw no change. Johnson et al.^36^ reported no significant changes in the severe cognitive impairment profile.

The three studies with mixed results^36^, ^44^, ^53^ included the three with the shortest durations: 12 weeks, 24 weeks, and 12 weeks, respectively. In contrast, those with positive results ranged from 20 weeks to 3 years in duration.^38^, ^39^, ^40^, ^42^ Of interest, the study with the consistently lowest daily dose of 3 mg throughout the whole intervention (24 weeks) reported significant improvements on the International Classification of Functioning, Disability and Health (ICF).^39^

### 3.5 Epigallocatechin gallate antioxidant (EGCG)

Epigallocatechin gallate (or EGCG) is a disease‐modifying antioxidant found in green tea and black tea.^31^, ^56^ EGCG may reduce amyloid beta plaques.^56^ One study^31^ examined the prevention of cognitive decline in young adults (mean = 23 years). EGCG (600 mg/day or 800 mg/day based on weight) was compared to placebo control over 1 year. Both groups also received cognitive training.^31^ Although executive function, Adaptive Behavior Assessment System II, and memory improved significantly in the experimental group, the placebo control reported no change.

### 3.6 Scyllo‐inositol (ELND005)

Scyllo‐inositol (or ELND005) is a disease‐modifying dietary supplement that may reduce the formation of amyloid beta plaque.^47^ One study examined the prevention of decline in cognition in young adults associated with using ELND005, with null outcomes.^47^ Participants were randomized into the two experimental groups. They either received 250 mg either once or twice a day. The control group received a the placebo. No difference in the adaptive behavior scale (Vineland Adaptive Behavior Scale Third Edition [Vineland‐3]), Neuropsychiatric Inventory, Block Design Subtest, or the cognition scale (Rapid Assessment of Developmental Disabilities, Second Edition [RADD‐2]) was reported between the experimental and placebo groups.

### 3.7 Fluoxetine, sertraline, paroxetine

Fluoxetine, sertraline, and paroxetine are selective serotonin reuptake inhibitors (or SSRIs), which block the reuptake of serotonin from the synapse, thereby increasing the amount of serotonin available.^34^ This treatment focuses on improving the symptoms of AD. One study examined the treatment of AD ‐elated declines in cognition associated with using fluoxetine, sertraline, and paroxetine for middle‐age adults (mean = 44.3), with positive effects.^34^ This collection of case studies included a variety of dosages based on other medication use. Overall, these SSRIs improved social interaction and independence in activities of daily living, while decreasing outbursts, compulsive behaviors, agitation, and negative behaviors.

### 3.8 Memantine

Memantine is a disease‐modifying treatment^55^ that works as an *N*‐methyl‐d‐aspartate (NMDA) blocker, which reduces the persistent activation of NMDA receptors thought to contribute to AD.^52^ Two studies^26^, ^52^ examined the prevention of AD‐related symptoms in young adults. One study examined the treatment of AD in elderly adults. Three studies examined the changes in cognition associated with memantine.^26^, ^35^, ^52^ All studies utilized a slow increase of daily doses across the intervention from 5 mg/day to 10 mg/twice/day, depending on the week of the intervention.^26^, ^35^, ^52^ The length of the interventions ranged from 16 weeks^26^, ^52^ to 52 weeks.^35^ No significant improvements were demonstrated in cognitive functioning, adaptive behavior, memory, or attention.

### 3.9 Nicotine (transdermal patch)

Nicotine is a symptom‐modifying drug that may help to improve attention, and has been demonstrating effectiveness in individuals with AD.^50^ One study examined the prevention of AD‐related decline in cognition associated with nicotine^50^ in young adults. Participants with and without DS (no control group) used a transdermal, single‐application 5 mg nicotine patch for 2 h. Significant improvements on the Digit‐Symbol Substitution Test subtest of the neuropsychological test were reported for the DS group compared to the non‐DS group.

### 3.10 Simvastatin

Simvastatin is a disease‐modifying statin used to lower low‐density lipoprotein cholesterol and may reduce amyloid beta protein in the brain.^30^, ^57^ One study examined the prevention of AD‐related decline in cognition associated with simvastatin, with positive effects in older adults.^30^ The experimental group took 40 mg/day for 12 months. Significant improvements in memory from Memory Object Test   in individuals with disabilities test were reported compared to a placebo control group.

### 3.11 Trazodone

Trazodone is a symptom‐modifying serotonin modulator, which inhibits serotonin transporter and serotonin type 2 receptors, thereby increasing available serotonin. It is used to treat depression, anxiety, and aggression. One case study examined the treatment of AD‐related decline in cognition associated with Trazodone in a 58‐year‐old man.^33^ The daily dose across the trial, starting with 100 mg and increasing to 200 mg for 70 days during two treatment phases. The number of minutes of aggression recorded reduced significantly from the baseline phase to the first treatment phase. There was an increase in aggression during the no‐treatment phase and a decrease during the second treatment phase.

### 3.12 Vitamin E

Vitamin E is a symptom‐modifying vitamin with antioxidant properties that may improve cognitive functioning.^49^, ^58^ One study examined the treatment of AD‐related decline in cognition associated with vitamin E in older adults with null effects.^49^ The experimental group used 670 mg, two times a day for 3 years. No significant improvements on the Brief Praxis Test were reported for the experimental group compared to a placebo control group.

### 3.13 ACI‐24 vaccine

ACI‐24 is a disease‐modifying vaccine that works to treat the misfolded AB proteins.^51^ One study examined the use of the ACI‐24 in the prevention of cognitive decline with null effects.^51^ Two experimental groups were given 300 and 1000 µg, respectively, seven times across 48 weeks. They were monitored for 96 weeks to assess the safety and tolerability of the vaccine. No significant improvements in reaction time, Paired Associative Learning, or adaptive behavior were reported for the experimental groups compared to the placebo control group.

### 3.14 Non‐pharmacological

Of the seven non‐pharmacological studies, three different intervention types were used: exercise (*n = *5), environmental (*n = *1), and cognitive training (*n = *1), these studies can be found in Table 2. A total of 225 individuals with DS were included.^27^, ^28^, ^29^, ^32^, ^41^, ^45^, ^48^ The number of participants ranged from age 18^27^ to 60.^32^ Five articles focused on prevention through improving or protecting from loss of cognitive function. One article included treatment of diagnosed AD/dementia. This group consisted of RCT (*n = *1), NRCT (*n = *4), and 2 group pre‐pos (*n = *1). The length of intervention ranged from one session^27^, ^28^, ^29^ to 3 years^32^.

**TABLE 2:** Non‐pharmacological intervention.

Authors	Risk of bias	Treatment or prevention	Participants	Mean ages (SD)	Intervention	Research design	Study dose or duration (time/session session/week total weeks)	Dependent variables	Summary of findings
McGlinchey et al., (2019)^41^	Low	Prevention	*N =* 40 Intervention: *N = 20*, *n *= 11 Female Control: *N* = 20, *n* = 15 Female	Intervention: *M =* 36.9 (5.65) Years Control: *M = *36.9 (5.9) Years	Cognitive Training: Online cognitive training program that included 12 games that targeted executive function	Quasi‐experimental (pre/post intervention and between groups) design with partial crossover for 8‐week intervention	20 min/day 5 days/week 8 weeks	Tower of London Weigl Card Sorting Scramble Boxes Dog & Cat Stroop Spatial reversal	Exp: Improvements to Cats and Dogs Stroop and Tower of London, Weigl Card Sorting. Scrambles boxes showed a trend toward improvements but did not reach significance. No significant difference in scores for spatial reversal. Control: No significant changes.
Chen & Ringenbach (2016)^27^	Medium	Prevention	*N = *18 Low‐Intensity: *N = 6* High‐Intensity: *N =* 6 Control: *N =* 6	Low‐Intensity: *M =* 23.70 (4.56) High‐Intensity: *M* = 22.10 (5.12) Control: *M* = 19.11 (3.00)	Exercise: Treadmill walking at 50‐75% or 75‐85% of predicted maximum heart rate Control: Watch a video	NRCT, 3 Groups: Pre‐Post	20 min/session 1 session total	Dimensional change card sort Test Knock Tap Test Choice Response Time	Moderate intensity group: performed significantly faster compared to the attentional control group on Informational processing speed. No change on the Attentional Shifting, Dimensional Change Card Sorting test. Significant improvement on Knock Tap Test. High intensity group: no change on Attentional Shifting. processing or Dimensional Change Card Sorting test. Significant improvement on Knock Tap Test. Control: No significant changes.
Chen et al., (2015)^28^	Medium	Prevention	*N =* 20 Attentional Group *N* = 10, *n* = 7 males Exercise Group *N* = 10, *n* = 8 males	*N = *20 Attentional Group *M = *20.58 (5.74) Exercise Group *M = *23.45 (4.86)	Exercise: Treadmill walking at 50%–75% of predicted maximum heart rate Control: Watch a video	NRCT, 2 group Pre‐Post	20 min/session 1 session total	Dimensional Change Card Sort Test Knock Tap Test Choice response time	Exp: Improvements on knock‐tap test (inhibition). No change to choice‐response time test or dimensional change card sort test. Control: No significant changes.
Chen & Ringenbach (2019)^29^	Low	Prevention	*N =* 26 Attentional Group *N *= 10, *n* = 7 male Low‐intensity Group *N *= 10, *n *= 8 male High‐intensity Group *N *= 8, *n* = 5 male	Attentional Group *M = 20.58 (5*.74) Low‐intensity Group *M* =* 2*1.42 *(5.46)* High‐intensity Group M = 22.70 (5.*69*)	Exercise: Treadmill walking at 50‐69% or 70‐85% of predicted maximum heart rate Control: Watch a video	NRCT, 3 Groups Pre‐Post:	20 min/session 1 session total	Phonetic Fluency Semantic Fluency Verbal Fluency	Exp Low Intensity: Improvements to semantic fluency. No changes to phonetic fluency and verbal fluency total. Exp high intensity: No changes to semantic fluency, phonetic fluency and verbal fluency total. Control: No changes to semantic fluency, phonetic fluency and verbal fluency total.
Ptomey et al., (2018)^45^	Low	Prevention	*N =* 27 PA once/week group: *N = 14*, *n *= 8 male PA twice/week group: *N* = 13, *n* = 8 male	PA once/week group *M = 29.9 (*7.5) PA twice/week group *M *= 25.8 (6.7)	Exercise: Aerobic based exercise class 1 or 2 times a week delivered via video conferencing	2 Groups Pre‐post	30 min/sessions 1 or 2 sessions/week 12 weeks	CANTAB‐ attention task Paired Associated Learning Reaction time	Exp 1/week: significant changes to memory. No other improvements. Exp 2/week: significant changes to memory. More improvements than 1/week. No other improvements.
Ringenbach et al., (2023)^48^	Medium	Prevention	*N =* 24 Assisted Cycle (ACT) Group *N = 12* Voluntary Cycle (VC Group *N = 10* Control Group N = 2	ACT Group: *M = 38.0(*8.81) VC Group: *M *= 36.2(9.27) Control Group *M* = 52.9(1.88)	ACT group pedaling rate was set to 35% greater than the participant's preferred pedaling rate. VC group operated in standard mode in which the motor does not provide any assistance with pedaling, and cadence and resistance were voluntarily selected by the participant. NC group participants were asked to maintain their current level of activity throughout the study period	RCT	30 minute/session 3 sessions/week 8 weeks	Self‐Efficacy Exercise Perception Questionnaires	ACT: treading towards improvement in self‐efficacy. VC: Improvement in self‐efficacy. Control: No differences.
De Vreese et al., (2012)^32^	High	Treatment	*N =* 60 Special Care Unit: *N* = 14, *n* = 7 Females Day Care: *N* = 22, *n* = 11 Females Nursing Home: *N* = 24, *n* = 12 Females	SCU: *M =* 53.2(6.9) DC: *M = *55.2(7.5) NH: *M = *51.9(5.5)	Changing environment: Special Care Unit: Specialized residential home with monitoring Day care: Day time care only centers Nursing Home: Standard nursing care	NRCT: 3 groups:	3 Years	Dementia questionnaire for intellectual disabilities sum of cognitive scores	SCU: improvements in short term memory, long term, language, DMR. DC: No improvements. NH: No improvements.

Abbreviations: Case, case studies; Exp, experimental group; NRCT, non‐randomized controlled trial; RCT, randomized controlled trial; SD, standard deviation

### 3.15 Exercise

Five studies examined the prevention of declines in attention, response time, fluency, and memory associated with exercise interventions in participants ranging from 18–58+ years.^27^, ^28^, ^29^, ^45^, ^48^ Three studies included a single 20‐min session of treadmill walking at a percentage of the participant's maximum heart rate.^27^, ^28^, ^29^ In these studies, the treadmill started at 0.5 mph and increased to 2.0 mph during warm‐up. Afterwards, the speed would stay between 2.0 and 3.0 mph, with the inclined increased by 2.5% every 4 min. Two of these studies included three groups: control, low‐intensity, and high‐intensity groups based on heart rate.^27^, ^28^ All three of the walking studies utilized attentional controls who watched a video.^27^, ^28^, ^29^ Significant improvements in information processing^27^ and inhibition^28^ were reported for the walking groups compared to the attention control groups.

One study examined changes in self‐efficacy following a cycling intervention that consisted of 30‐min of cycling three times a week for 8 weeks.^48^ This study had three groups. The assisted cycling therapy group pedaled at a rate set 35% higher than the participants’ preferred rate, with a motor assisting the rider. The voluntary cycling group pedaled at the preferred rate with no assistance. The control group was asked to maintain their current level of activity. Significant improvements to self‐efficacy were reported for the voluntary cycling group; no improvements were observed for the other two groups.

One study examined cognitive function following a 30‐min aerobic‐based exercise class with two groups: once or twice a week for 12 weeks.^45^ Both groups showed significant improvements in the memory assessment (Paired Associated Learning), with the twice weekly group exhibiting a greater improvement than the once weekly group.

### 3.16 Cognitive training

One study examined the prevention of decline in executive function following an 8‐week cognitive training intervention in middle‐aged adults (age m = 36.9).^41^ The cognitive training was an online‐based game that targeted executive functioning, and The consisted of 12 online games that practiced memory, processing, planning, attention, and problem‐solving. The intervention was 5 days a week and 20 min per session for 8 weeks. Participants were quasi‐randomized into the experimental and control groups. Significant improvements in inhibition, working memory, and planning were reported for the cognitive training group compared to the control group.

### 3.17 Environmental change

One study examined the treatment of memory loss over 3 years following an environmental change intervention in elderly adults.^32^ Three different resistance types were assessed for their effects on AD‐related outcomes over 3 years. Participants resided at a specialized residential home with monitoring, a daytime care‐only center, or standard nursing home. Significantly better long‐ and short‐term memory and language functions were reported for those living in a specialized residential home compared to both other groups.

### 3.18 Meta‐analysis

**FIGURE 4:**
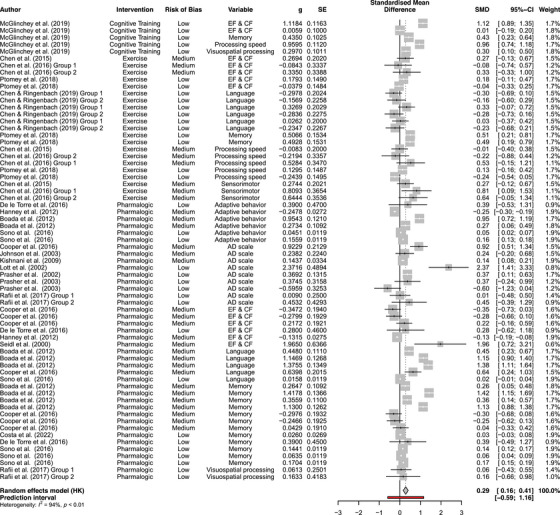
Meta‐analysis.

Figure 4 is a forest plot depicting the overall meta‐effect sizes and the individual study effect sizes by experimental and control groups, and organized by outcome measure. A meta‐analysis was used to assess differences in cognitive outcomes between the experimental and control groups (Figure 4). An assessment of variation among studies revealed high heterogeneity among effects (*I* ^2^ *= *93.8%, 95% confidence interval [CI]: 92.8–94.7). Overall, the interventions resulted in a small but significant effect on cognitive and behavior outcomes (*t* (66) = 4.67, *p *< 0.0001, *d *= 0.29, 95% CI: 0.16–0.40).

Follow‐up meta‐regressions were conducted to examine treatment type (pharmacological, non‐pharmacological), dose, study risk of bias (low, medium, high), and outcome type (insert outcome types here). For treatment types, there was no significant between‐group effect (*p *= 0.39), suggesting that both pharmacological and non‐pharmacological interventions had similar effects. There was no significant effect of risk of bias (*p *= 0.15), suggesting that studies with low, medium, and high risk of bias had similar effects. For outcome type, there were no significant between‐group effects (*p *= 0.64), suggesting that the interventions had similar effects on all cognitive and behavioral domains.

## 4 DISCUSSION

This is the first comprehensive review of AD‐related outcomes of interventions and treatments for individuals with DS. Two previous reviews qualitatively evaluated pharmacological interventions for AD in adults with DS.^19^ This study replicates and extends those reviews in several important ways: (1) the inclusion of non‐pharmacological studies; (2) conducting both qualitative synthesis and a meta‐analysis; and (3) examining differences in effect sizes with respect to key mediating variables such as treatment type, dose, risk of bias, and outcome type. The meta‐analysis revealed that pharmacological and non‐pharmacological interventions effectively improve AD‐related cognitive and behavioral outcomes. However, additional research is needed to replicate and extend these interventions, given the overall high study heterogeneity reported here.

Since the creation of the Investigation of cooccurring conditions across the lifespan to understand down syndrome (INCLUDE) workgroup in 2017,^59^ greater consistency in outcome assessments has been observed. The assessments that are used in the Trial‐Ready Cohort‐Down Syndrome (TRC‐DS), which was developed specifically for clinical trials for AD in persons with DS includes the Cued Recall Test, Down Syndrome Mental Status Examination (DS‐MSE), Kaufman Brief Intelligence Test, Second Edition (KBIT‐2), and Stroop Dog and Cat Task. In addition, caregiver‐reported dementia‐related problems and adaptive behavior are assessed using the National Task Group Early Detection Screen for Dementia (NTG‐EDSD) and Vineland Adaptive Behavior Scale 3 (Vineland‐3; informant version), respectively. Heterogeneity due to the use of different outcome assessments may be due, in part, to the inclusion of 19 studies published before harmonized cognitive outcomes were established in 2017.^22^ As more studies examine the prevention and treatment of AD in persons with DS, it is essential that consistent and validated outcomes measures are employed to enable more accurate comparisons across studies and to determine which interventions work best for which individuals and when.

It is important to note that of the 26 studies that were included in the qualitative synthesis, 17 reported data to enable their inclusion in the quantitative synthesis (meta‐analysis and meta‐regression). It is possible that the lack of differences in effect sizes reported in the meta‐analysis may be driven by heterogeneity in the number of studies per drug type and a greater number of donepezil trials. Indeed, donepezil is the most studied pharmacological treatment for AD in persons with and without DS. For patients with AD without DS, significant cognitive and global functioning^60^ improvements have been observed. The qualitative synthesis included seven donepezil studies, of which four reported significant improvements in individuals with DS (Kondoh et al.^38^; Kondoh et al.^39^; Lott et al.^40^; Prasher et al., 2003b). Greater effects were observed for longer treatment periods (20 weeks to 3 years). Five of these studies were included in the quantitative synthesis. With these promising results in mind, additional donepezil trials are needed to determine if there are differences in efficacy for patients with DS. Additionally, trials with donepezil in conjunction with non‐pharmacological intervention should be conducted.

Although many other pharmacological interventions were included in the qualitative and quantitative synthesis, the effects of these other drug or supplement types are less well documented. Here, we focus our discussion on the pharmacological interventions that positively impact cognitive and behavioral outcomes in participants with DS. One study reported that simvastatin improved memory in individuals with DS.^30^ This is consistent with reports that simvastatin can help prevent AD in non‐DS populations.^61^ These promising results from Cooper et al.^30^, coupled with the successful use in the non‐DS populations, indicate the need to further the line of study on simvastatin in DS populations.

Similarly, one study examined a single application of a transdermic nicotine patch and reported improved cognitive functioning.^50^ Although these results are promising, the efficacy of nicotine as a treatment for AD is still unclear,^62^ and there are adverse side effects that warrant further investigation for individuals with DS.^62^, ^63^

One study examined EGCG with cognitive training. Greater improvements were reported for memory and executive functioning, indicating that the combination of the two interventions was more effective than cognitive training alone. Future pharmacological studies should examine the potential additive or multiplicative effects when pharmacological treatments are paired with non‐pharmacological interventions.

Depression frequently co‐occurs with AD and may be an early symptom of the disease.^64^, ^65^, ^66^ The two studies examining antidepressants (fluoxetine, sertraline, paroxetine, and trazodone) focused on improving depression and related behavioral symptoms: aggression,^33^ agitation,^34^ impaired social interactions,^34^ negative compulsive behavior,^34^ hallucinations,^34^ and lack of activities of daily living.^34^ These studies did not examine executive function or cognition directly. However, both studies reported marked improvements in depression and related behavioral outcomes. Improvements in these behavioral areas may reduce the overall care workload for caregivers (Brzezińska et al.^64^; He et al.^65^) and improve quality of life for patients (He et al.^65^). Future studies are needed to examine the long‐term effects of treating the symptoms of AD.

Very few pharmacological interventions were not effective in improving cognitive or behavioral outcomes in persons with DS. It is surprising that some pharmacological interventions that have been found to be effective in patients with AD without DS, including acetylcarnitine^67^ and memantine,^52^ were not effective in the studies included here with participants with DS (Preasher et al.^45^, Johnson et al.^36^, Kishnani et al.,^53^ Rafii et al., 2007, Boada et al.^26^, Costa et al.^52^, Hanney et al.^35^). Future studies are needed to determine if the dose, duration, or participant characteristics affect treatment effects.

All seven non‐pharmacological studies report significant improvements. Three of the exercise studies examined a single session of 20–30 min,^27^, ^28^, ^29^ suggesting that even small intervention doses may be effective and feasible for this population. However, additional research is needed to replicate and extend these studies to evaluate the long‐term impact on other key AD‐related cognitive domains (e.g., memory, cognition, and language). Another exercise study (add citation in) included a longer intervention duration, was delivered virtually, and compared one and two sessions per week. No group differences were observed, providing additional evidence that exercise, even in a virtual format, is effective and feasible for this population. The one study that examined online cognitive training also found significant improvement in cognitive functioning. Again, these results are encouraging as online formats may be more scalable by reducing barriers to in‐person participation.

### 4.1 Limitations and future directions

Significant heterogeneity in cognitive assessments across studies has made it challenging to compare intervention effects directly. Standardized, sensitive assessments are needed to enable meaningful, apples‐to‐apples comparisons. Previous research has indicated the weakness of research in DS in using appropriate and validated outcomes to study cognition.^22^

Although stratification of treatment effects based on premorbid intelligence quotient (IQ) and AD stage would be very informative, the articles examined here did not consistently provide information about IQ or AD stage. Regarding AD stage, recent studies have suggested that people with DS are at AD stage 0 at birth because of their elevated genetic risk for AD and the presence of AD biomarkers early during development.^68^

Another recent study examined the efficacy of several disease‐modifying drugs in improving cognitive outcomes in children and adolescents with DS.^69^ Although the studies included in that review are outside the scope of this article (focused on adults), the results suggest that intervening at early stages of development before the onset of AD symptoms is a potentially impactful future direction. Long‐term follow‐up with pediatric patients is needed to determine if early intervention changes the trajectory of AD in adulthood.

The present study provides additional evidence of the need to harmonize study designs, outcomes assessments, and inclusion/exclusion criteria to enable direct comparison across studies and treatments. Future studies may investigate whether higher doses, longer intervention durations, or other individual characteristics (e.g., level of intellectual disability or AD stage) affect treatment outcomes. These interventions and participant characteristics are critical to determine which interventions are most effective for which individuals. Moreover, additional studies are needed to characterize potential safety, side effects, and dosing differences for persons with DS. For example, Rafii (2022) noted that in the study by Costa et al.^52^, the plasma concentrations of memantine were lower than the therapeutic range (i.e., 0.37 µmol/L vs 0.5–1.0 µmol/L). These results suggest different pharmacokinetics and pharmacodynamics that have important implications on dosing, tolerability, and safety for persons with DS.

Although pharmacological interventions like donepezil have shown promise, additional studies are required to determine their long‐term efficacy. Likewise, non‐pharmacological interventions, such as exercise and cognitive training, have demonstrated potential benefits, but further research is necessary to assess their sustained impact on memory, language, and executive function.

Two studies, Gedye^33^ and Geldmacher et al.,^34^ address commonly reported aggression and depression. These may be considered co‐morbidities rather than symptoms. Future research should clarify whether these should be treated separately from AD. Preliminary findings suggest that combining pharmacological and non‐pharmacological approaches may enhance cognitive and behavioral outcomes. Future studies should explore the potential multiplicative effects of these combination therapies. Of interest, some pharmacological treatments effective for AD patients without DS did not yield the same benefits for individuals with DS. This discrepancy warrants further investigation into factors such as dosage, treatment duration, genetic variability, and underlying biological differences. A more personalized approach may be necessary to optimize therapeutic outcomes.

The success of online cognitive training and virtual exercise interventions highlights the feasibility of remote delivery methods. Future research should explore ways to scale these interventions while ensuring accessibility for individuals with DS and their caregivers. Developing cost‐effective and widely available treatment strategies could significantly improve the quality of care and support for this population.

## CONFLICT OF INTEREST STATEMENT

None of the authors have any conflicts of interest to disclose. Any author disclosures are available in the Supporting Information.

## CONSENT STATEMENT

The consent statement is not required as this work is a review.

## Supporting information



Supporting Information

## References

[1] De Graaf G , Buckley F , Skotko BG . Estimation of the number of people with Down syndrome in the United States. Genet Med. 2017;19(4):439‐447. 27608174 10.1038/gim.2016.127

[2] Yang Q , Rasmussen SA , Friedman J . Mortality associated with Down's syndrome in the USA from 1983 to 1997: a population‐based study. Lancet. 2002;359(9311):1019‐1025. 11937181 10.1016/s0140-6736(02)08092-3

[3] Presson AP , Partyka G , Jensen KM , et al. Current estimate of Down syndrome population prevalence in the United States. J Pediatr. 2013;163(4):1163‐1168. 23885965 10.1016/j.jpeds.2013.06.013 PMC4445685

[4] Iulita MF , Garzón Chavez D , Klitgaard Christensen M , et al. Association of Alzheimer disease with life expectancy in people with down syndrome. JAMA Netw Open. 2022;5(5):e2212910‐e2212910. doi:10.1001/jamanetworkopen.2022.12910 35604690 PMC9127560

[5] McCarron M , McCallion P , Reilly E , Dunne P , Carroll R , Mulryan N . A prospective 20‐year longitudinal follow‐up of dementia in persons with Down syndrome. J Intellect Disabil Res. 2017;61(9):843‐852. 28664561 10.1111/jir.12390

[6] Snyder HM , Bain LJ , Brickman AM , et al. Further understanding the connection between Alzheimer's disease and Down syndrome. Alzheimers Dement. 2020;16(7):1065‐1077. 32544310 10.1002/alz.12112 PMC8865308

[7] Rubenstein E , Tewolde S , Michals A , et al. Alzheimer dementia among individuals with down syndrome. JAMA Netw Open. 2024;7(9):e2435018. doi:10.1001/jamanetworkopen.2024.35018 39312235 PMC11420697

[8] Fortea J , Zaman SH , Hartley S , Rafii MS , Head E , Carmona‐Iragui M . Alzheimer's disease associated with Down syndrome: a genetic form of dementia. Lancet Neurol. 2021;20(11):930‐942. 34687637 10.1016/S1474-4422(21)00245-3 PMC9387748

[9] Hithersay R , Startin CM , Hamburg S , et al. Association of dementia with mortality among adults with Down syndrome older than 35 years. JAMA Neurol. 2019;76(2):152‐160. 30452522 10.1001/jamaneurol.2018.3616 PMC6439956

[10] Rafii MS , Fortea J . Down syndrome in a new era for Alzheimer disease. JAMA. 2023;330(22):2157‐2158. doi:10.1001/jama.2023.22924 37991807 PMC11324235

[11] Ahmad F , Karan A , Sharma R , et al. Evolving therapeutic interventions for the management and treatment of Alzheimer's disease. Ageing Res Rev. 2024;95:102229. 38364913 10.1016/j.arr.2024.102229

[12] Ali H , Imtiaz H , Rehman AU , et al. An Applied Artificial Intelligence Technique for Early‐Stage Alzheimer's Disease Prediction. IEEE; 2024:1‐8.

[13] Duan Y , Lu L , Chen J , et al. Psychosocial interventions for Alzheimer's disease cognitive symptoms: a Bayesian network meta‐analysis. BMC Geriatr. 2018;18(1):1‐11. doi:10.1186/s12877-018-0864-6 30086714 PMC6081912

[14] Ren L , Zhang Q , Zhou J , Wang X , Zhu D , Chen X . Leveraging diverse regulated cell death patterns to identify diagnosis biomarkers for Alzheimer's disease. J Prev Alzheimer's Dis. 2024;11(6):1775‐1788. 39559889 10.14283/jpad.2024.119 PMC11573840

[15] Yu T‐W , Lane H‐Y , Lin C‐H . Novel therapeutic approaches for Alzheimer's disease: an updated review. Int J Mol Sci. 2021;22(15):8208. 34360973 10.3390/ijms22158208 PMC8348485

[16] Xiang C , Zhang Y . Comparison of cognitive intervention strategies for individuals with Alzheimer's disease: a systematic review and network meta‐analysis. Neuropsychol Rev. 2024;34(2):402‐416. 36929474 10.1007/s11065-023-09584-5 PMC11166762

[17] Bahrami S , Momtazmanesh S , Rezaei N . Music therapy for Alzheimer's disease management: a narrative review. Egypt J Neurol Psychiatry Neurosurg. 2024;60(1):66.

[18] Cummings J , Zhou Y , Lee G , Zhong K , Fonseca J , Cheng F . Alzheimer's disease drug development pipeline: 2024. Alzheimer's Dement: Transl Res Clin Interv. 2024;10(2):e12465. 10.1002/trc2.12465 PMC11040692 38659717

[19] Keeling LA , Spiridigliozzi GA , Hart SJ , Baker JA , Jones HN , Kishnani PS . Challenges in measuring the effects of pharmacological interventions on cognitive and adaptive functioning in individuals with Down syndrome: a systematic review. Am J Med Genet A. 2017;173(11):3058‐3066. doi:10.1002/ajmg.a.38416 28857390

[20] Livingstone N , Hanratty J , McShane R , Macdonald G . Pharmacological interventions for cognitive decline in people with Down syndrome. Cochrane Database Syst Rev. 2015(10):CD011546. doi:10.1002/14651858.CD011546.pub2 26513128 PMC8763347

[21] Rafii MS . Improving memory and cognition in individuals with Down syndrome. CNS Drugs. 2016;30(7):567‐573. 27272473 10.1007/s40263-016-0353-4

[22] Esbensen AJ , Hooper SR , Fidler D , et al. Outcome measures for clinical trials in Down syndrome. Am J Intellect Dev Disabil. 2017;122(3):247‐281. 28452584 10.1352/1944-7558-122.3.247 PMC5424621

[23] Page MJ , McKenzie JE , Bossuyt PM , et al. The PRISMA 2020 statement: an updated guideline for reporting systematic reviews. BMJ. 2021;372. 10.1136/bmj.n71 PMC8005924 33782057

[24] Sterne JA , Savović J , Page MJ , et al. RoB 2: a revised tool for assessing risk of bias in randomised trials. BMJ. 2019;366:l4898. 31462531 10.1136/bmj.l4898

[25] Sterne JA , Hernán MA , Reeves BC , et al. ROBINS‐I: a tool for assessing risk of bias in non‐randomised studies of interventions. BMJ. 2016;355:i4919. 27733354 10.1136/bmj.i4919 PMC5062054

[26] Boada R , Hutaff‐Lee C , Schrader A , et al. Antagonism of NMDA receptors as a potential treatment for Down syndrome: a pilot randomized controlled trial. Transl Psychiatry. 2012;2:e141. doi:10.1038/tp.2012.66 22806212 PMC3410988

[27] Chen CCJJ , Ringenbach SDR . Dose‐response relationship between intensity of exercise and cognitive performance in individuals with Down syndrome: a preliminary study. J Intellect Disabil Res: JIDR. 2016;60(6):606‐614. doi:10.1111/jir.12258 26923820

[28] Chen CCJJ , Ringenbach SDR , Crews D , Kulinna PH , Amazeen EL . The association between a single bout of moderate physical activity and executive function in young adults with Down syndrome: a preliminary study. J Intellect Disabil Res: JIDR. 2015;59(7):589‐598. doi:10.1111/jir.12163 25171600

[29] Chen CC , Ringenbach SDR . The effect of acute exercise on the performance of verbal fluency in adolescents and young adults with Down syndrome: a pilot study. J Intellect Disabil Res. 2019;63(6):614‐623. doi:10.1111/jir.12603 30811082

[30] Cooper S‐A , Ademola T , Caslake M , et al. Towards onset prevention of cognition decline in adults with Down syndrome (The TOP‐COG study): a pilot randomised controlled trial. Trials. 2016;17:1‐16. doi:10.1186/s13063-016-1370-9 27473843 PMC4966871

[31] de la Torre R , de Sola S , Hernandez G , et al. Safety and efficacy of cognitive training plus epigallocatechin‐3‐gallate in young adults with Down's syndrome (TESDAD): a double‐blind, randomised, placebo‐controlled, phase 2 trial. Lancet Neurol. 2016;15(8):801‐810. doi:10.1016/S1474-4422(16)30034-5 27302362

[32] De Vreese LP , Mantesso U , De Bastiani E , Weger E , Marangoni AC , Gomiero T . Impact of dementia‐derived nonpharmacological intervention procedures on cognition and behavior in older adults with intellectual disabilities: a 3‐year follow‐up study. J Policy Pract Intellect Disabil. 2012;9(2):92‐102. doi:10.1111/j.1741-1130.2012.00344.x

[33] Gedye A . Serotonergic treatment for aggression in a down's syndrome adult showing signs of Alzheimer's disease. J Ment Defic Res. 1991;35(Pt 3):247‐258. doi:10.1111/j.1365-2788.1991.tb01058.x 1833553

[34] Geldmacher DS , Lerner AJ , Voci JM , Noelker EA , Somple LC , Whitehouse PJ . Treatment of functional decline in adults with Down syndrome using selective serotonin‐reuptake inhibitor drugs. J Geriatr Psychiatry Neurol. 1997;10(3):99‐104. doi:10.1177/089198879701000302 9322131

[35] Hanney M , Prasher V , Williams N , et al. Memantine for dementia in adults older than 40 years with Down's syndrome (MEADOWS): a randomised, double‐blind, placebo‐controlled trial. Lancet (Lond Engl). 2012;379(9815):528‐536. doi:10.1016/S0140-6736(11)61676-0 22236802

[36] Johnson N , Fahey C , Chicoine B , Chong G , Gitelman D . Effects of donepezil on cognitive functioning in Down syndrome. Am J Ment Retard. 2003;108(6):367‐372. doi:10.1352/0895-8017(2003)108<367:EODOCF>2.0.CO;2 14561111

[37] Kishnani PS , Spiridigliozzi GA , Heller JH , Sullivan JA , Doraiswamy PM , Krishnan KR . Donepezil for Down's syndrome. American Psychiatric Publishing, Inc.; 2001:143‐143. 10.1176/appi.ajp.158.1.143 11136652

[38] Kondoh T , Amamoto N , Doi T , et al. Dramatic improvement in Down syndrome‐associated cognitive impairment with donepezil. Ann Pharmacother. 2005;39(3):563‐566. doi:10.1345/aph.1E427 15701776

[39] Kondoh T , Kanno A , Itoh H , et al. Donepezil significantly improves abilities in daily lives of female Down syndrome patients with severe cognitive impairment: a 24‐week randomized, double‐blind, placebo‐controlled trial. Int J Psychiatry Med. 2011;41(1):71‐89. doi:10.2190/PM.41.1.g 21495523

[40] Lott IT , Osann K , Doran E , Nelson L . Down syndrome and Alzheimer disease: response to donepezil. Arch Neurol. 2002;59(7):1133‐1136. 12117361 10.1001/archneur.59.7.1133

[41] McGlinchey E , McCarron M , Holland A , McCallion P . Examining the effects of computerised cognitive training on levels of executive function in adults with Down syndrome. J Intellect Disabil Res. 2019;63(9):1137‐1150. doi:10.1111/jir.12626 31062455

[42] Prasher VP , Adams C , Holder R . Long term safety and efficacy of donepezil in the treatment of dementia in Alzheimer's disease in adults with Down syndrome: open label study. Article. Int J Geriatr Psychiatry. 2003;18(6):549. 12789681 10.1002/gps.859

[43] Prasher VP , Fung N , Adams C . Rivastigmine in the treatment of dementia in Alzheimer's disease in adults with Down syndrome. Int J Geriatr Psychiatry. 2005;20(5):496‐497. doi:10.1002/gps.1306 15852458

[44] Prasher VP , Huxley A , Haque MS . A 24‐week, double‐blind, placebo‐controlled trial of donepezil in patients with Down syndrome and Alzheimer's disease–pilot study. Int J Geriatr Psychiatry. 2002;17(3):270‐278. doi:10.1002/gps.587 11921156

[45] Ptomey LT , Szabo AN , Willis EA , et al. Changes in cognitive function after a 12‐week exercise intervention in adults with Down syndrome. Disabil health j. 2018;11(3):486‐490. doi:10.1016/j.dhjo.2018.02.003 29501470 PMC6005720

[46] Pueschel SM . The effect of acetyl‐L‐carnitine administration on persons with down syndrome. Res Dev Disabil: Multidiscip J. 2006;27(6):599‐604. doi:10.1016/j.ridd.2004.07.009 16621446

[47] Rafii MS , Skotko BG , McDonough ME , et al. A randomized, double‐blind, placebo‐controlled, phase II study of oral ELND005 (scyllo‐Inositol) in young adults with down syndrome without dementia. J Alzheimer's Dis. 2017;58(2):401‐411. doi:10.3233/JAD-160965 28453471 PMC5777855

[48] Ringenbach SDR , Arnold NE , Tucker K , et al. Assisted Cycle Therapy (ACT) improved self‐efficacy and exercise perception in middle‐age adults with down syndrome. article. Brain Sci (2076‐3425). 2023;13(12):1719. doi:10.3390/brainsci13121719 38137167 PMC10741653

[49] Sano M , Aisen PS , Andrews HF , Tsai W‐Y , Lai F , Dalton AJ . Vitamin E in aging persons with Down syndrome: a randomized, placebo‐controlled clinical trial. Neurology. 2016;86(22):2071‐2076. doi:10.1212/WNL.0000000000002714 27164691 PMC4891209

[50] Seidl R , Tiefenthaler M , Hauser E , Lubec G . Effects of transdermal nicotine on cognitive performance in down's syndrome. Article. Lancet. 2000;356(9239):1409. doi:10.1016/S0140-6736(00)02848-8 11052587

[51] Rafii MS , Sol O , Mobley WC , et al. Safety, tolerability, and immunogenicity of the ACI‐24 vaccine in adults with down syndrome: a phase 1b randomized clinical trial. JAMA Neurol. 2022;79(6):565‐574. doi:10.1001/jamaneurol.2022.0983 35532913 PMC9086937

[52] Costa ACS , Brandão AC , Boada R , et al. Safety, efficacy, and tolerability of memantine for cognitive and adaptive outcome measures in adolescents and young adults with Down syndrome: a randomised, double‐blind, placebo‐controlled phase 2 trial. Lancet Neurol. 2022;21(1):31‐41. doi:10.1016/S1474-4422(21)00369-0 34942135

[53] Kishnani PS , Sommer BR , Handen BL , et al. The efficacy, safety, and tolerability of donepezil for the treatment of young adults with Down syndrome. Am J Med Genet A. 2009;149A(8):1641‐1654. doi:10.1002/ajmg.a.32953 19606472

[54] Sano M , Aisen PS , Andrews HF , et al. Vitamin E in aging persons with Down syndrome: a randomized, placebo‐controlled clinical trial. Neurology. 2016;86(22):2071‐2076. doi:10.1212/WNL.0000000000002714 27164691 PMC4891209

[55] Monteiro AR , Barbosa DJ , Remião F , Silva R . Alzheimer's disease: insights and new prospects in disease pathophysiology, biomarkers and disease‐modifying drugs. Biochem Pharmacol. 2023;211:115522. 36996971 10.1016/j.bcp.2023.115522

[56] Guedj F , Sébrié C , Rivals I , et al. Green tea polyphenols rescue of brain defects induced by overexpression of DYRK1A. PloS one. 2009;4(2):e4606. 19242551 10.1371/journal.pone.0004606 PMC2645681

[57] Li L , Cao D , Kim H , Lester R , Fukuchi K . Simvastatin enhances learning and memory independent of amyloid load in mice. Ann Neurol: Off J Am Neurol Assoc Child Neurol Soc. 2006;60(6):729‐739. 10.1002/ana.21053 17192930

[58] Dysken MW , Sano M , Asthana S , et al. Effect of vitamin E and memantine on functional decline in Alzheimer disease: the TEAM‐AD VA cooperative randomized trial. Jama. 2014;311(1):33‐44. 24381967 10.1001/jama.2013.282834 PMC4109898

[59] Baumer NT , Becker ML , Capone GT , et al. Conducting clinical trials in persons with Down syndrome: summary from the NIH INCLUDE Down syndrome clinical trials readiness working group. J Neurodev Disord. 2022;14(1):22. 35321660 10.1186/s11689-022-09435-z PMC8942061

[60] Adlimoghaddam A , Neuendorff M , Roy B , Albensi BC . A review of clinical treatment considerations of donepezil in severe Alzheimer's disease. CNS Neurosci Ther. 2018;24(10):876‐888. 30058285 10.1111/cns.13035 PMC6489741

[61] Dhakal S , Macreadie IG . Simvastatin, its antimicrobial activity and its prevention of Alzheimer's disease. Microorganisms. 2024;12(6):1133. 38930515 10.3390/microorganisms12061133 PMC11205914

[62] Hoskin JL , Al‐Hasan Y , Sabbagh MN . Nicotinic acetylcholine receptor agonists for the treatment of Alzheimer's dementia: an update. Nicotine Tob Res. 2019;21(3):370‐376. 30137524 10.1093/ntr/nty116 PMC6379052

[63] Alhowail A . Molecular insights into the benefits of nicotine on memory and cognition. Mol Med Rep. 2021;23(6):1‐6. 10.3892/mmr.2021.12037 PMC8025477 33786606

[64] Brzezińska A , Bourke J , Rivera‐Hernández R , Tsolaki M , Woźniak J , Kaźmierski J . Depression in dementia or dementia in depression? Systematic review of studies and hypotheses. Curr Alzheimer Res. 2020;17(1):16‐28. 32065103 10.2174/1567205017666200217104114

[65] He Y , Li H , Huang J , et al. Efficacy of antidepressant drugs in the treatment of depression in Alzheimer disease patients: a systematic review and network meta‐analysis. J Psychopharmacol. 2021;35(8):901‐909. 34238048 10.1177/02698811211030181

[66] Orgeta V , Qazi A , Spector A , Orrell M . Psychological treatments for depression and anxiety in dementia and mild cognitive impairment: systematic review and meta‐analysis. Br J Psychiatry. 2015;207(4):293‐298. 26429684 10.1192/bjp.bp.114.148130 PMC4589662

[67] Pennisi M , Lanza G , Cantone M , et al. Acetyl‐L‐carnitine in dementia and other cognitive disorders: a critical update. Nutrients. 2020;12(5):1389. 32408706 10.3390/nu12051389 PMC7284336

[68] Jack Jr CR , Andrews JS , Beach TG , et al. Revised criteria for diagnosis and staging of Alzheimer's disease: Alzheimer's association workgroup. Alzheimer's Dement. 2024;20(8):5143‐5169. 38934362 10.1002/alz.13859 PMC11350039

[69] Lorenzon N , Musoles Lleó J , Turrisi F , Gomis González M , De La Torre R , Dierssen M . State of the art therapy for Down syndrome. Dev Med Child Neurol. 2023;65(7):870‐884. 36692980 10.1111/dmcn.15517

